# A Case of Refractory Hiccups and Numbness as the Initial Presentation of Multiple Sclerosis

**DOI:** 10.7759/cureus.81774

**Published:** 2025-04-05

**Authors:** Kouichi Asahi

**Affiliations:** 1 Internal Medicine and Pediatrics, Kohokuekimae Ohisama Clinic, Tokyo, JPN; 2 General Medicine and Radiology, Dokkyo Medical University Saitama Medical Center, Saitama, JPN

**Keywords:** brainstem lesion, multiple sclerosis, neuroimmunology, neuromyelitis optica spectrum disorder, refractory hiccups

## Abstract

Multiple sclerosis (MS) is a chronic autoimmune demyelinating disease of the central nervous system (CNS), commonly presenting with optic neuritis, motor dysfunction, or sensory disturbances. However, intractable hiccups as an initial manifestation are exceedingly rare. Early recognition of such atypical symptoms is crucial for timely diagnosis and intervention.

A 19-year-old woman presented with intractable hiccups and progressive numbness affecting her right arm and trunk. Neurological examination revealed right upper limb weakness, sensory impairment from the clavicle to the upper abdomen, and hyperreflexia in the right lower limb. Brain MRI demonstrated a hyperintense lesion in the dorsal medulla on axial T2-weighted imaging, with mild enhancement on contrast-enhanced T1-weighted imaging. Cervical spine MRI showed a high-intensity lesion extending from C2 to C6. Serological tests for aquaporin-4 (AQP4)-IgG and myelin oligodendrocyte glycoprotein (MOG)-IgG were negative. Based on her clinical and imaging findings, a diagnosis of MS was established according to the 2017 McDonald criteria. She was treated with high-dose corticosteroids followed by ofatumumab (Kesimpta®), a B-cell depletion therapy, leading to significant symptom improvement.

This case underscores the need to recognize intractable hiccups as a potential brainstem symptom of MS. Furthermore, we discuss the challenges in diagnosing oligoclonal band-negative MS and the potential role of alternative biomarkers such as neurofilament light chain (NfL). Early diagnosis and appropriate management can improve patient outcomes, particularly in cases involving the brainstem.

## Introduction

Multiple sclerosis (MS) is a chronic immune-mediated neuroinflammatory disease characterized by demyelination and axonal injury within the central nervous system (CNS) [1]. It typically affects young adults and commonly presents with optic neuritis, limb weakness, diplopia, or sensory disturbances [2]. In contrast, neuromyelitis optica spectrum disorder (NMOSD) and myelin oligodendrocyte glycoprotein antibody-associated disease (MOGAD) may also affect the CNS but are immunologically distinct entities with different therapeutic implications [3].

Intractable hiccups and numbness as the initial manifestation of MS are extremely rare. These symptoms are more frequently associated with NMOSD, particularly when the dorsal medulla or area postrema is involved [4]. Nevertheless, MS can also involve the brainstem and produce similar symptoms. A few case reports have described MS presenting with intractable hiccups or nausea, indicating the need for careful differential diagnosis in early stages [5].

## Case presentation

A 19-year-old woman initially developed intermittent hiccups, which gradually worsened and became intractable, eventually leading to nausea and significant difficulty with oral intake. Around the same time, she experienced progressive numbness in the right arm and trunk, as well as mild weakness in the right hand. Neurological examination revealed mild weakness in the right upper limb, with muscle strength graded as 4/5 in the biceps, triceps, and wrist extensors, and 3/5 in the thenar and hypothenar muscles. Strength in the lower limbs was normal. Sensory examination showed reduced touch and pain sensation on the right side, extending from the clavicle to the umbilicus. Reflex testing demonstrated hyperreflexia in the right lower limb, with brisk patellar and Achilles reflexes. No abnormalities were observed in cranial nerve function. However, persistent, intractable hiccups suggested the involvement of autonomic pathways.

**Figure 1:**
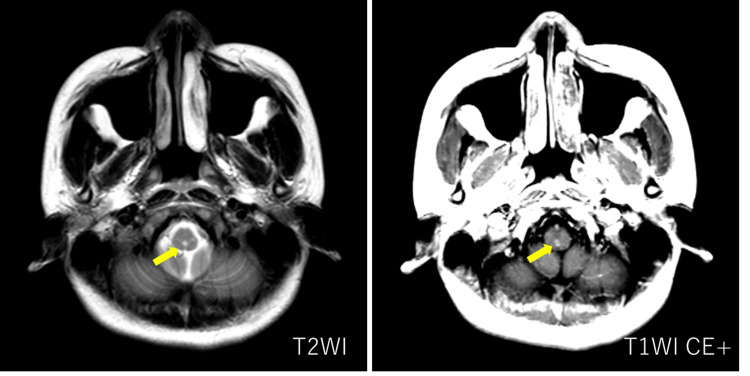
Axial brain MRI at the medullary level Axial T2-weighted MRI showing a hyperintense lesion in the dorsal medulla, with mild enhancement on contrast-enhanced T1-weighted imaging (yellow arrow).

**Figure 2:**
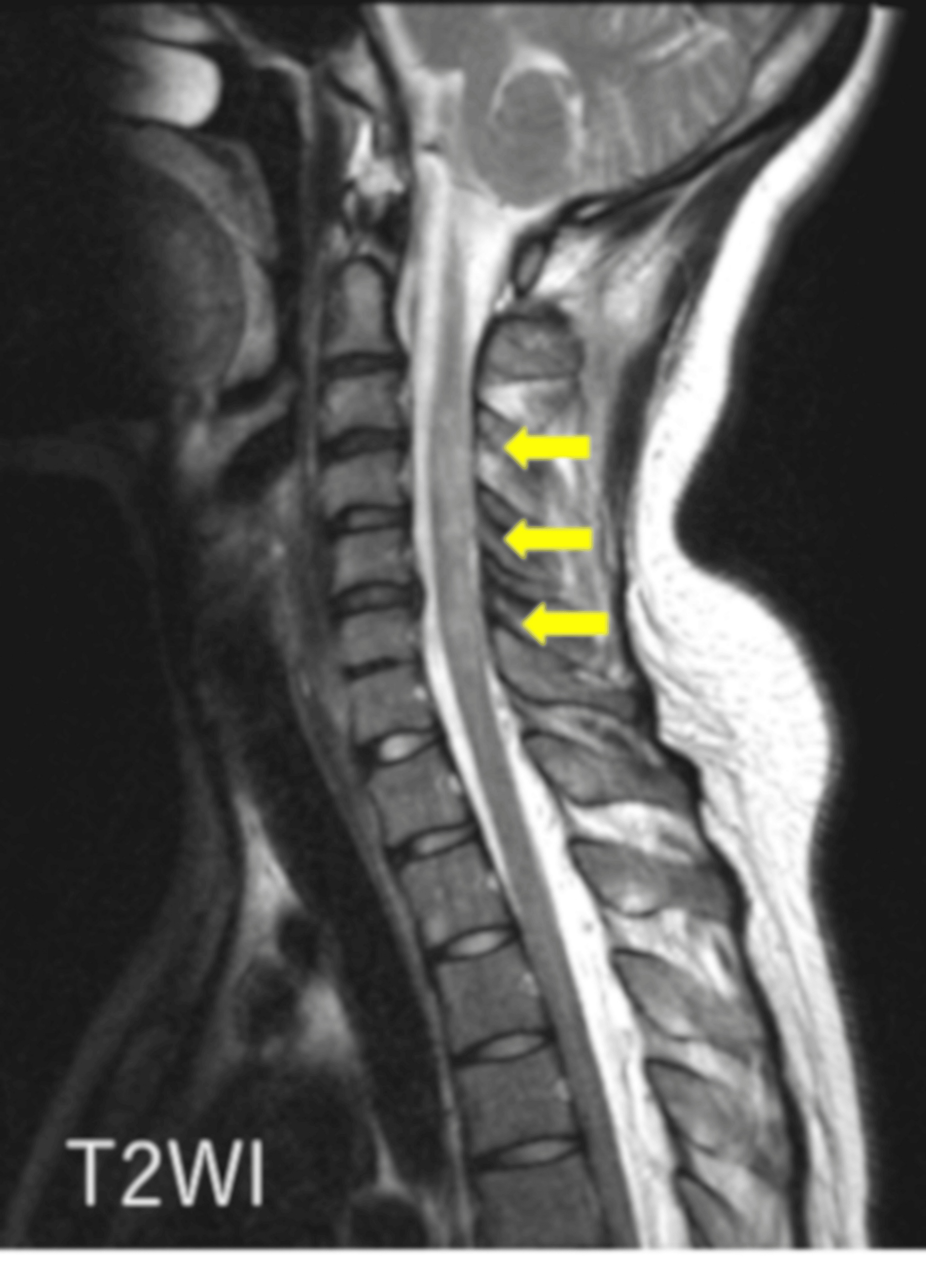
Sagittal non-contrast MRI of the cervical spine Sagittal T2-weighted cervical spine MRI demonstrating a hyperintense lesion extending from C2 to C6 (yellow arrows).

**Figure 3:**
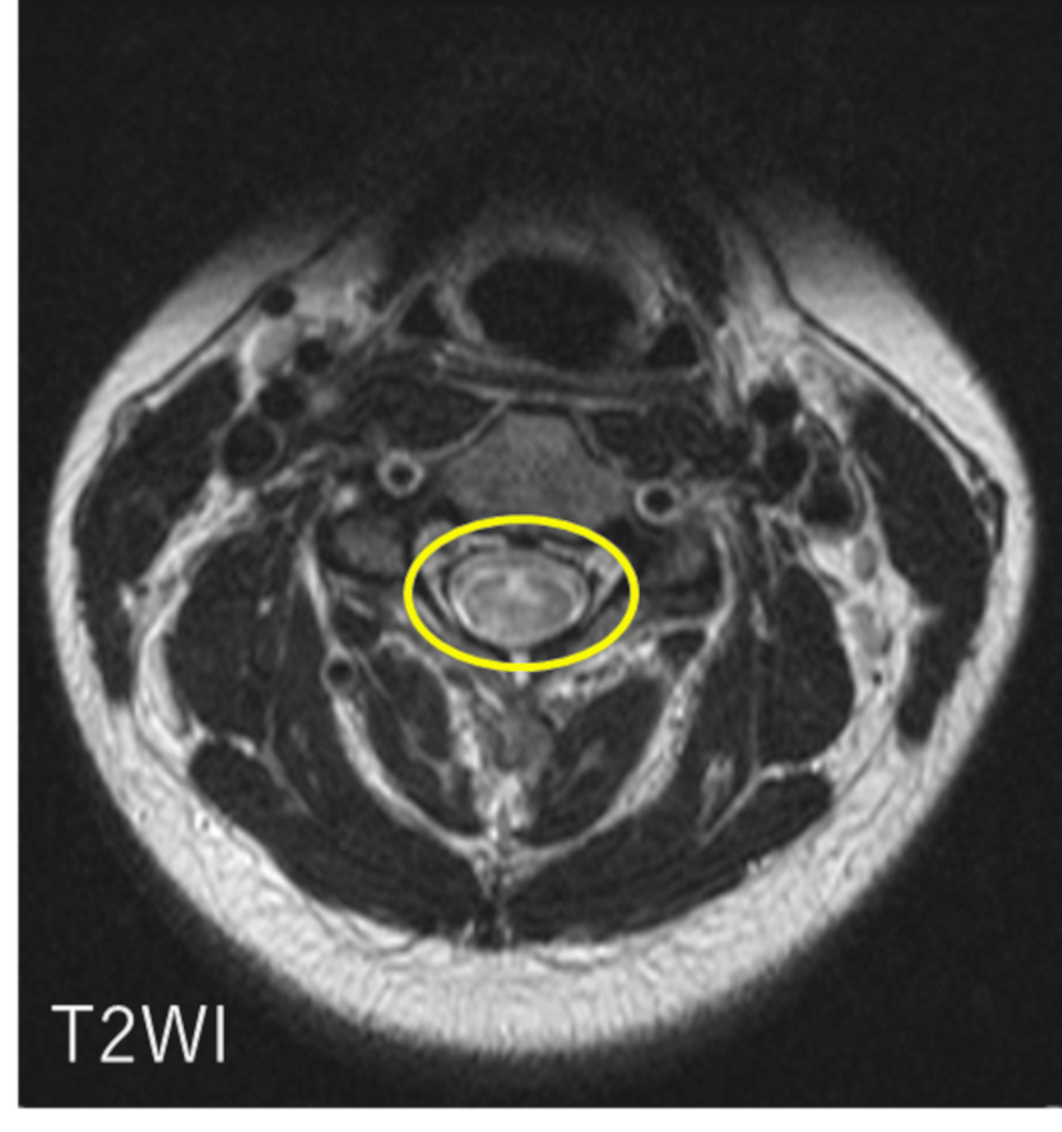
Axial non-contrast MRI of the cervical spine Axial T2-weighted MRI at the C3 level showing right-sided spinal cord hyperintensity (yellow circle).

Given the presence of intractable hiccups and a brainstem lesion, NMOSD was initially suspected. To evaluate for a possible longitudinally extensive lesion in the cervical spinal cord, additional MRI was performed. A brain MRI revealed a hyperintense lesion in the dorsal medulla on axial T2-weighted images, with mild enhancement observed on contrast-enhanced T1-weighted imaging (Figure 1). Sagittal T2-weighted cervical spine MRI demonstrated a hyperintense lesion extending from C2 to C6 (Figure 2). Axial T2-weighted MRI at the C3 level showed right-sided spinal cord hyperintensity (Figure 3).

Cerebrospinal fluid analysis showed a mild elevation in white blood cell count (7/μL), normal protein and glucose levels, and a negative result for oligoclonal bands. The IgG index was within normal range. Serological tests for aquaporin-4 (AQP4-IgG) and myelin oligodendrocyte glycoprotein (MOG-IgG) antibodies were also negative. Based on the clinical and radiological findings, the patient was diagnosed with multiple sclerosis according to the 2017 McDonald criteria [6]. The presence of lesions in the brainstem and cervical spinal cord indicated dissemination in space, while her history suggested dissemination in time. After completing three days of high-dose intravenous methylprednisolone (1 g/day), the patient was started on monthly subcutaneous ofatumumab (Kesimpta®). Over a six-month follow-up period, she has maintained clinical improvement without relapse or new neurological symptoms [7].

## Discussion

Intractable hiccups and nausea are well-documented symptoms in NMOSD, occurring in approximately 17% of cases, often due to area postrema involvement. However, this case highlights that MS can also involve the dorsal medulla and present with similar symptoms. Key differentiating factors are as follows: MS: Patchy or multifocal brainstem lesions, negative AQP4-IgG, good response to corticosteroids, and B-cell depletion therapy; NMOSD: Area postrema lesions, longitudinally extensive spinal cord lesions, AQP4-IgG positivity, and poor steroid response.

Another challenge in this case was the absence of oligoclonal bands (OCBs) in the CSF, as up to 10% of MS patients may be OCB-negative [8]. Emerging biomarkers such as neurofilament light chain (NfL) and glial fibrillary acidic protein (GFAP) may provide additional diagnostic value in these cases [9].

## Conclusions

This case highlights the need to recognize intractable hiccups as a potential initial symptom of MS. Additionally, it underscores the importance of considering alternative biomarkers in OCB-negative MS cases. Early diagnosis and appropriate management are essential for optimal patient outcomes, particularly in brainstem-involved MS.
